# Redetermination of poly[μ-chlorido-hepta­chlorido-μ_3_-l-proline-μ_2_-l-proline-tetra­mercury(II)]

**DOI:** 10.1107/S160053680802196X

**Published:** 2008-07-19

**Authors:** D. Kalaiselvi, R. Mohan Kumar, R. Jayavel

**Affiliations:** a Crystal Growth Centre, Anna University, Chennai 600025, India; b Department of Physics, Presidency College, Chennai 600005, India

## Abstract

The asymmetric unit of the title compound, [Hg_4_Cl_8_(C_5_H_9_NO_2_)_2_]_*n*_, consists of four HgCl_2_ units and two L-proline ligands in the zwitterionic form. In each HgCl_2_ unit, the Hg^II^ ion is strongly bonded to two Cl atoms, and the Hg^II^ ions in two of the HgCl_2_ units are chelated by O atoms of two l-proline ligands, with one strong and one weak Hg—O bond. In the crystal structure, HgCl_2_ and L-proline units are linked to form an extended chain along the *a* axis. The chain structure is further stabilized by N—H⋯Cl hydrogen bonds, and the chains are arranged in layers parallel to the *ab* plane. The structure of the title compound was originally determined by Ehsan, Malik & Haider [(1996). *J. Banglad. Acad. Sci.* **20**, 175] but no three-dimensional coordinates are available.

## Related literature

For related literature, see: Janczak & Luger (1997 ▶); Jiang & Fang (1999 ▶); Kurtz & Perry (1968 ▶); Long (1995 ▶); McL Mathieson & Welsh (1952 ▶); Nockemann & Meyer (2002 ▶); Padmanabhan *et al.* (1995 ▶); Pandiarajan *et al.* (2002*a* ▶,*b* ▶); Schaffers & Keszler (1993 ▶); Subha Nandhini *et al.* (2001 ▶); Tedmann *et al.* (2004 ▶); Yukawa *et al.* (1982 ▶, 1983 ▶, 1985 ▶); Ehsan *et al.* (1996 ▶).
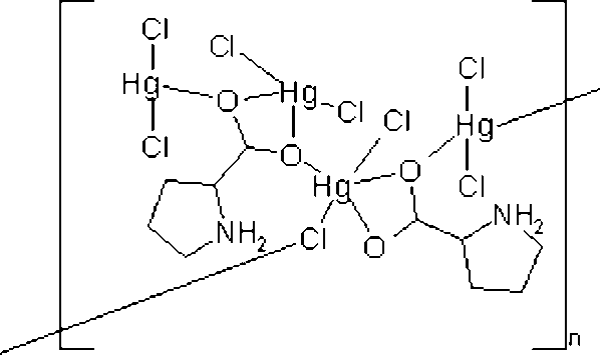


## Experimental

### 

#### Crystal data

[Hg_4_Cl_8_(C_5_H_9_NO_2_)_2_]
*M* *_r_* = 1316.23
Triclinic, 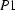

*a* = 7.2742 (4) Å
*b* = 9.4472 (5) Å
*c* = 10.4767 (6) Å
α = 108.621 (3)°
β = 107.260 (2)°
γ = 97.353 (2)°
*V* = 631.51 (6) Å^3^
*Z* = 1
Mo *K*α radiation
μ = 25.10 mm^−1^
*T* = 293 (2) K
0.20 × 0.10 × 0.10 mm

#### Data collection

Bruker Kappa APEXII area-detector diffractometer
Absorption correction: multi-scan (Blessing, 1995 ▶) *T*_min_ = 0.082, *T*_max_ = 0.188 (expected range = 0.035–0.081)
10896 measured reflections
3956 independent reflections
3773 reflections with *I* > 2σ(*I*)
*R*_int_ = 0.044

#### Refinement

*R*[*F* ^2^ > 2σ(*F* ^2^)] = 0.037
*wR*(*F* ^2^) = 0.111
*S* = 1.03
3956 reflections
255 parameters
21 restraints
H-atom parameters constrained
Δρ_max_ = 1.75 e Å^−3^
Δρ_min_ = −2.51 e Å^−3^
Absolute structure: Flack (1983 ▶), 1736 Friedel pairs
Flack parameter: 0.057 (16)

### 

Data collection: *APEX2* (Bruker, 2004 ▶); cell refinement: *APEX2*; data reduction: *SAINT* (Bruker, 2004 ▶); program(s) used to solve structure: *SIR92* (Altomare *et al.*, 1993 ▶); program(s) used to refine structure: *SHELXL97* (Sheldrick, 2008 ▶); molecular graphics: *ORTEP-3* (Farrugia, 1997 ▶); software used to prepare material for publication: *SHELXL97* and *PLATON* (Spek, 2003 ▶).

## Supplementary Material

Crystal structure: contains datablocks I, global. DOI: 10.1107/S160053680802196X/lh2640sup1.cif

Structure factors: contains datablocks I. DOI: 10.1107/S160053680802196X/lh2640Isup2.hkl

Additional supplementary materials:  crystallographic information; 3D view; checkCIF report

## Figures and Tables

**Table 1:** Selected bond lengths (Å)

O4—Hg4	2.888 (10)
O4—Hg3	2.828 (11)
O2—Hg2	2.869 (13)
O1—Hg1	2.564 (11)
O1—Hg2	2.566 (12)
O3—Hg3	2.486 (13)
O3—Hg2	2.634 (12)
Cl1—Hg1	2.326 (6)
Cl2—Hg1	2.276 (6)
Cl3—Hg2	2.323 (6)
Cl4—Hg2	2.337 (6)
Cl5—Hg3	2.316 (6)
Cl6—Hg3	2.304 (6)
Cl7—Hg4	2.300 (6)
Cl8—Hg4	2.255 (7)
Hg1—Cl3^i^	3.009 (6)

Symmetry code: (i) 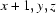.

**Table 2:** Hydrogen-bond geometry (Å, °)

*D*—H⋯*A*	*D*—H	H⋯*A*	*D*⋯*A*	*D*—H⋯*A*
N1—H1*A*⋯Cl7^i^	0.90	2.63	3.282 (14)	130
N1—H1*B*⋯Cl6^i^	0.90	2.40	3.290 (16)	167
N2—H2*A*⋯Cl4^ii^	0.90	2.40	3.267 (15)	163
N2—H2*B*⋯Cl5^ii^	0.90	2.60	3.234 (15)	128

Symmetry codes: (i) 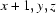; (ii) 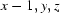.

**Fig. 1.**
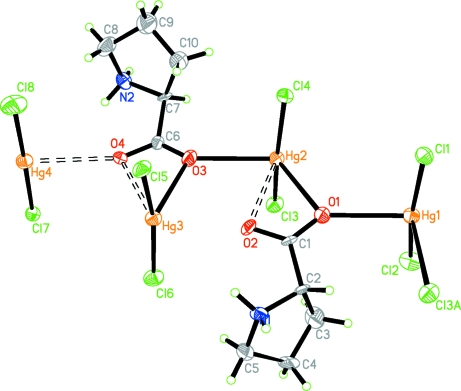
The asymmetric unit of the title compound. Displacement ellipsoids are drawn at the 30% probability level. Atom Cl3A is generated by the symmetry operation (1+ x, y, z). Dashed bonds indicate weak interactions.

**Fig. 2.**
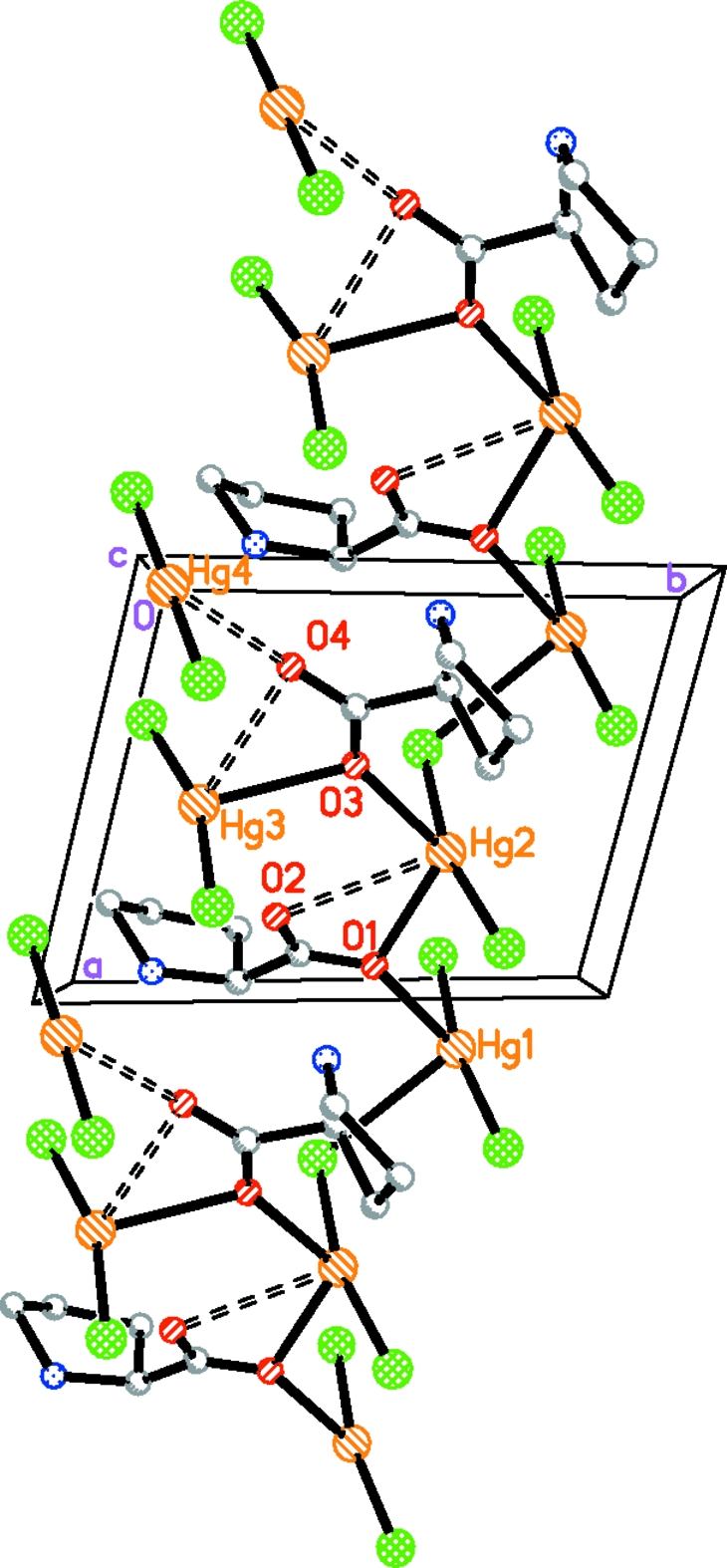
Part of an extended chain running along the a axis. Dashed bonds indicate weak interactions.

**Fig. 3.**
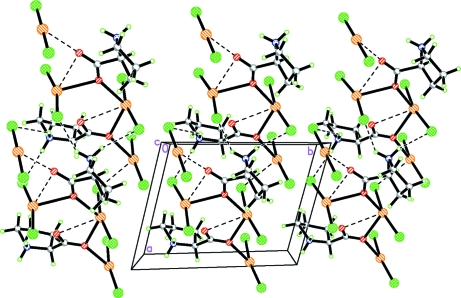
The crystal packing of the title compound, viewed along the c axis. Hydrogen bonds are shown as dashed lines.`

## supplementary crystallographic information

### Comment

During the last few years, organic non-linear optical (NLO) crystals have attracted much interest due to their superior properties over inorganic NLO materials, such as higher susceptibility, faster response and the capability of designing components on the molecular level. However, unlike inorganic NLO crystals, they have not come into wide use, owing to drawbacks such as the difficulty of growing large size perfect single crystals and poor physicochemical stability. Under these circumstances, crystals of metal-organic materials with NLO effects have been developed which are expected not only to retain high NLO effects, but also to minimize some of the shortcomings of pure organic crystals; in other words, they have the advantages of both organic and inorganic crystals in terms of their physicochemical properties. This approach has resulted in their practical use in frequency-doubling of laser radiation (Long, 1995; Jiang & Fang, 1999). The crystal structure of L-proline monohydrate (Janczak & Luger, 1997), DL-proline monohydrate (Padmanabhan *et al.*, 1995), L-prolinium tartrate (Subha Nandhini *et al.*,2001), bis (L-proline) hydrogen (1+) perchlorate (Pandiarajan *et al.*,2002*a*), bis (L-proline) hydrogen nitrate (Pandiarajan *et al.*, 2002*b*), L-alanine cadmium chloride (Schaffers & Keszler, 1993), dichloro(4-hydroxy-L-proline)cadmium(II) (Yukawa *et al.*, 1982), dichloro(L-proline)cadmium(II) hydrate (Yukawa *et al.*, 1983), dichlorobis(L-proline)Zinc(II) (Yukawa *et al.*, 1985) and bis-DL-prolinatocopper(II)dihydrate (McL Mathieson & Welsh, 1952) have been reported. The present study reports the crystal structure of the title salt, a complex of L-proline with mercury chloride. The second harmonic generation (SHG) effect of the crystals was measured by the powder SHG technique (Kurtz & Perry, 1968) and was found to be 2.5 times that of potassium dihydrogen phosphate crystals.

The asymmetric unit consists of four HgCl_2_ units and two L-proline zwitterions (Fig.1). In each HgCl_2_ units the metal atom is strongly bonded to two Cl atoms, with Hg—Cl distances in the range 2.255 (7) Å–2.337 (6) Å. These distances are comparable with those observed for ammonium mercury (II) dichloride nitrate (Nockemann & Meyer, 2002) and (2,2-bipyridine *N*,*N*'-dioxide-k_2_O,*O*')dichloro-mercury(II) (Tedmann *et al.*, 2004). Metal atoms in two HgCl_2_ units (Hg2 and Hg3) are also chelated by carboxylate O atoms of two L-proline ligands, with one strong and one weak Hg—O bonds [Hg2—O1 2.566 (12) Å, Hg2—O2 2.869 (13) Å, Hg3—O3 2.486 (13) Å and Hg3—O4 2.828 (13) Å]. These distances are comparable with those observed for ammonium mercury (II) dichloride nitrate (Nockemann & Meyer, 2002). The two HgCl_2_L units (L is L-proline) are linked *via* Hg2—O3 bond [Hg2—O3 2.634 (12) Å]. Of the remaining two HgCl_2_units, one unit (Hg1) is bonded to atom O1 of the adjacent L-proline ligand and atom Cl3 in the adjacent unit cell [Hg1···O1 2.564 (11) Å and Hg1—Cl3(1 + *x*,*y*,*z*) 3.009 (7) Å], and the other unit is weakly bonded with atom O4 [Hg4—O4 2.888 (12) Å]. The geometry around metal atoms Hg1, Hg2 and Hg3 is nearly linear as a result of constraints imposed by chelation [Cl2—Hg1—Cl1 168.25 (18)°, Cl3—Hg2—Cl4 166.19 (18)° and Cl6—Hg3—Cl5 163.51 (18)°] whereas that around atom Hg4 is linear [Cl8—Hg4—Cl7 178.5 (3)°].

In the crystal structure, the HgCl_2_ and L-proline units are linked to form an extended chain along the *a* axis (Fig.2). The chain structure is further strengthened by N—H···Cl hydrogen bonds (Table 2). The polymeric chains are arranged into layers parallel to the *ab* plane (Fig.3). The structure of the title compound was originally determined by Ehsan *et al.* (1996) but no three-dimensional coordinates are available.

### Experimental

The title compound was crystallized at room temperature by slow evaporation of an aqueous solution of L-proline and mercury(II) chloride in a stoichimetric ratio of 1:2.

### Refinement

The large anisotropic displacement parameters of atoms C3, C8 and C9 suggested disorder in five-membered rings. But attempts to refine the structure with a disorder model did not improve these parameters. Hence, during the final cycles of refinement the U^ij^ components of atoms C3, C8 and C9 were restrained to approximate isotropic behaviour. The unresolved disorder resulted in poor precision on C—C bond lengths. H atoms were placed in idealized positions and allowed to ride on their parent atoms, with N—H = 0.90 Å and C—H = 0.97 or 0.98 Å and *U*_iso_(H) = 1.2*U*_eq_(C,*N*).

### Crystal data

**Table:**

[Hg_4_Cl_8_(C_5_H_9_NO_2_)_2_]	*Z* = 1
*M**_r_* = 1316.23	*F*_000_ = 580
Triclinic, *P*1	*D*_x_ = 3.461 Mg m^−^^3^
Hall symbol: P 1	Mo *K*α radiation λ = 0.71073 Å
*a* = 7.2742 (4) Å	Cell parameters from 5619 reflections
*b* = 9.4472 (5) Å	θ = 2.4–35.5º
*c* = 10.4767 (6) Å	µ = 25.10 mm^−^^1^
α = 108.621 (3)º	*T* = 293 (2) K
β = 107.260 (2)º	Plate, pale brown
γ = 97.353 (2)º	0.20 × 0.10 × 0.10 mm
*V* = 631.51 (6) Å^3^	

### Data collection

**Table:**

Bruker Kappa APEXII area-detector diffractometer	3956 independent reflections
Radiation source: fine-focus sealed tube	3773 reflections with *I* > 2σ(*I*)
Monochromator: graphite	*R*_int_ = 0.044
*T* = 293(2) K	θ_max_ = 25.0º
ω and φ scans	θ_min_ = 2.4º
Absorption correction: multi-scan(Blessing, 1995)	*h* = −6→8
*T*_min_ = 0.082, *T*_max_ = 0.188	*k* = −11→11
10896 measured reflections	*l* = −12→12

### Refinement

**Table:**

Refinement on *F*^2^	Hydrogen site location: inferred from neighbouring sites
Least-squares matrix: full	H-atom parameters constrained
*R*[*F*^2^ > 2σ(*F*^2^)] = 0.037	*w* = 1/[σ^2^(*F*_o_^2^) + (0.0781*P*)^2^ + 1.0577*P*] where *P* = (*F*_o_^2^ + 2*F*_c_^2^)/3
*wR*(*F*^2^) = 0.111	(Δ/σ)_max_ = 0.001
*S* = 1.03	Δρ_max_ = 1.75 e Å^−^^3^
3956 reflections	Δρ_min_ = −2.51 e Å^−^^3^
255 parameters	Extinction correction: SHELXL97 (Sheldrick, 2008), Fc^*^=kFc[1+0.001xFc^2^λ^3^/sin(2θ)]^-1/4^
21 restraints	Extinction coefficient: 0.0066 (5)
Primary atom site location: structure-invariant direct methods	Absolute structure: Flack (1983), 1736 Friedel pairs
Secondary atom site location: difference Fourier map	Flack parameter: 0.057 (16)

### Special details

**Table:**

Geometry. All e.s.d.'s (except the e.s.d. in the dihedral angle between two l.s. planes) are estimated using the full covariance matrix. The cell e.s.d.'s are taken into account individually in the estimation of e.s.d.'s in distances, angles and torsion angles; correlations between e.s.d.'s in cell parameters are only used when they are defined by crystal symmetry. An approximate (isotropic) treatment of cell e.s.d.'s is used for estimating e.s.d.'s involving l.s. planes.
Refinement. Refinement of *F*^2^ against ALL reflections. The weighted *R*-factor *wR* and goodness of fit *S* are based on *F*^2^, conventional *R*-factors *R* are based on *F*, with *F* set to zero for negative *F*^2^. The threshold expression of *F*^2^ > σ(*F*^2^) is used only for calculating *R*-factors(gt) *etc*. and is not relevant to the choice of reflections for refinement. *R*-factors based on *F*^2^ are statistically about twice as large as those based on *F*, and *R*- factors based on ALL data will be even larger.

### Fractional atomic coordinates and isotropic or equivalent isotropic displacement parameters (Å^2^)

**Table:**

	*x*	*y*	*z*	*U*_iso_*/*U*_eq_	
C1	0.899 (3)	0.4306 (18)	0.7785 (18)	0.028 (4)	
C2	0.969 (2)	0.3323 (15)	0.6707 (14)	0.029 (3)	
H2	1.1092	0.3762	0.6931	0.034*	
C3	0.849 (3)	0.307 (2)	0.515 (2)	0.061 (5)	
H3A	0.7262	0.3395	0.5091	0.073*	
H3B	0.9246	0.3647	0.4779	0.073*	
C4	0.805 (3)	0.135 (2)	0.4312 (19)	0.053 (5)	
H4A	0.6851	0.1011	0.3467	0.063*	
H4B	0.9145	0.1099	0.4004	0.063*	
C5	0.782 (3)	0.063 (2)	0.529 (2)	0.054 (5)	
H5A	0.8072	−0.0391	0.5014	0.065*	
H5B	0.6496	0.0542	0.5329	0.065*	
C6	0.330 (2)	0.4234 (17)	1.0096 (17)	0.029 (4)	
C7	0.286 (2)	0.5707 (15)	1.1024 (14)	0.031 (3)	
H7	0.2394	0.6301	1.0431	0.037*	
C8	0.224 (3)	0.559 (3)	1.308 (2)	0.066 (5)	
H8A	0.2775	0.4742	1.3230	0.079*	
H8B	0.1311	0.5775	1.3584	0.079*	
C9	0.385 (4)	0.700 (3)	1.359 (3)	0.074 (7)	
H9A	0.4888	0.7116	1.4480	0.089*	
H9B	0.3341	0.7919	1.3756	0.089*	
C10	0.463 (3)	0.673 (2)	1.237 (2)	0.059 (5)	
H10A	0.5709	0.6227	1.2514	0.070*	
H10B	0.5103	0.7701	1.2293	0.070*	
N1	0.942 (2)	0.1748 (13)	0.6775 (12)	0.040 (3)	
H1A	0.9010	0.1744	0.7504	0.048*	
H1B	1.0575	0.1460	0.6915	0.048*	
N2	0.1290 (17)	0.5265 (14)	1.1579 (15)	0.042 (3)	
H2A	0.0369	0.5809	1.1448	0.050*	
H2B	0.0685	0.4256	1.1103	0.050*	
O1	0.9293 (18)	0.5718 (13)	0.7961 (14)	0.044 (3)	
O2	0.8094 (19)	0.3750 (13)	0.8405 (12)	0.050 (3)	
O3	0.457 (2)	0.4477 (15)	0.9648 (17)	0.055 (4)	
O4	0.2390 (17)	0.2970 (11)	0.9976 (12)	0.039 (3)	
Cl1	1.3639 (8)	0.8860 (6)	0.9691 (6)	0.0403 (14)	
Cl2	0.9186 (9)	0.6904 (7)	0.5046 (7)	0.0569 (15)	
Cl3	0.4113 (9)	0.5596 (8)	0.6683 (7)	0.0463 (14)	
Cl4	0.8766 (8)	0.7849 (6)	1.1323 (6)	0.0388 (11)	
Cl5	0.7759 (8)	0.2678 (7)	1.1413 (6)	0.0430 (13)	
Cl6	0.3481 (8)	0.0587 (7)	0.6721 (6)	0.0504 (14)	
Cl7	−0.1508 (9)	−0.0454 (6)	0.8492 (6)	0.0408 (14)	
Cl8	0.2824 (12)	0.1691 (10)	1.3145 (8)	0.076 (2)	
Hg1	1.13721 (7)	0.76197 (5)	0.73403 (5)	0.0368 (2)	
Hg2	0.65755 (7)	0.64526 (5)	0.89544 (5)	0.0337 (2)	
Hg3	0.54733 (7)	0.19563 (5)	0.91009 (5)	0.0357 (2)	
Hg4	0.07001 (8)	0.06525 (6)	1.08346 (6)	0.0426 (2)	

### Atomic displacement parameters (Å^2^)

**Table:**

	*U*^11^	*U*^22^	*U*^33^	*U*^12^	*U*^13^	*U*^23^
C1	0.035 (9)	0.035 (8)	0.017 (8)	0.013 (6)	0.017 (7)	0.004 (7)
C2	0.038 (8)	0.030 (7)	0.021 (7)	0.013 (6)	0.019 (6)	0.005 (6)
C3	0.083 (9)	0.056 (8)	0.047 (8)	0.015 (6)	0.012 (6)	0.034 (7)
C4	0.077 (14)	0.041 (9)	0.014 (8)	0.002 (8)	0.007 (8)	−0.010 (7)
C5	0.072 (13)	0.036 (9)	0.044 (11)	−0.002 (8)	0.022 (10)	0.006 (8)
C6	0.024 (9)	0.034 (8)	0.025 (8)	0.002 (6)	0.007 (7)	0.012 (7)
C7	0.045 (9)	0.033 (7)	0.012 (6)	0.008 (6)	0.014 (6)	0.002 (6)
C8	0.059 (8)	0.086 (9)	0.060 (9)	0.010 (6)	0.039 (7)	0.024 (7)
C9	0.071 (10)	0.079 (10)	0.066 (10)	0.005 (7)	0.022 (8)	0.025 (8)
C10	0.036 (10)	0.072 (12)	0.044 (11)	−0.014 (8)	0.006 (8)	0.010 (9)
N1	0.073 (9)	0.030 (6)	0.022 (6)	0.017 (6)	0.017 (6)	0.012 (5)
N2	0.025 (6)	0.040 (6)	0.052 (9)	0.002 (5)	0.009 (6)	0.015 (6)
O1	0.055 (8)	0.034 (6)	0.052 (8)	0.012 (5)	0.030 (6)	0.016 (6)
O2	0.067 (8)	0.052 (7)	0.028 (6)	−0.007 (6)	0.029 (6)	0.008 (5)
O3	0.078 (9)	0.044 (7)	0.066 (10)	0.022 (6)	0.054 (8)	0.024 (7)
O4	0.052 (7)	0.026 (5)	0.032 (6)	0.000 (5)	0.018 (5)	0.004 (5)
Cl1	0.034 (3)	0.041 (3)	0.040 (3)	0.006 (2)	0.008 (2)	0.013 (2)
Cl2	0.054 (3)	0.057 (3)	0.049 (3)	0.021 (3)	0.003 (2)	0.017 (3)
Cl3	0.039 (3)	0.058 (3)	0.038 (3)	0.012 (2)	0.010 (2)	0.017 (2)
Cl4	0.044 (3)	0.034 (2)	0.032 (3)	0.005 (2)	0.012 (2)	0.008 (2)
Cl5	0.040 (3)	0.055 (3)	0.026 (3)	0.002 (2)	0.009 (2)	0.011 (2)
Cl6	0.045 (3)	0.059 (3)	0.034 (3)	0.008 (2)	0.007 (2)	0.008 (3)
Cl7	0.051 (3)	0.037 (3)	0.036 (3)	0.012 (2)	0.016 (2)	0.015 (2)
Cl8	0.063 (4)	0.093 (5)	0.038 (3)	0.005 (3)	−0.004 (3)	0.006 (3)
Hg1	0.0368 (4)	0.0374 (4)	0.0338 (4)	0.0094 (3)	0.0100 (3)	0.0127 (3)
Hg2	0.0300 (4)	0.0369 (4)	0.0325 (4)	0.0074 (3)	0.0099 (3)	0.0125 (3)
Hg3	0.0298 (4)	0.0387 (4)	0.0323 (4)	0.0050 (3)	0.0082 (3)	0.0097 (3)
Hg4	0.0438 (5)	0.0425 (4)	0.0349 (5)	0.0072 (3)	0.0095 (3)	0.0119 (4)

### Geometric parameters (Å, °)

**Table:**

C1—O2	1.224 (19)	C9—C10	1.51 (3)
C1—O1	1.267 (19)	C9—H9A	0.97
C1—C2	1.480 (19)	C9—H9B	0.97
C2—N1	1.502 (16)	C10—H10A	0.97
C2—C3	1.53 (2)	C10—H10B	0.97
C2—H2	0.98	N1—H1A	0.90
C3—C4	1.52 (2)	N1—H1B	0.90
C3—H3A	0.97	N2—H2A	0.90
C3—H3B	0.97	N2—H2B	0.90
C4—C5	1.44 (2)	O4—Hg4	2.888 (10)
C4—H4A	0.97	O4—Hg3	2.828 (11)
C4—H4B	0.97	O2—Hg2	2.869 (13)
C5—N1	1.57 (2)	O1—Hg1	2.564 (11)
C5—H5A	0.97	O1—Hg2	2.566 (12)
C5—H5B	0.97	O3—Hg3	2.486 (13)
C6—O3	1.18 (2)	O3—Hg2	2.634 (12)
C6—O4	1.236 (17)	Cl1—Hg1	2.326 (6)
C6—C7	1.56 (2)	Cl2—Hg1	2.276 (6)
C7—N2	1.495 (18)	Cl3—Hg2	2.323 (6)
C7—C10	1.52 (2)	Cl4—Hg2	2.337 (6)
C7—H7	0.98	Cl5—Hg3	2.316 (6)
C8—N2	1.43 (2)	Cl6—Hg3	2.304 (6)
C8—C9	1.49 (3)	Cl7—Hg4	2.300 (6)
C8—H8A	0.97	Cl8—Hg4	2.255 (7)
C8—H8B	0.97	Hg1—Cl3^i^	3.009 (6)
			
O2—C1—O1	123.4 (15)	C9—C10—H10B	110.8
O2—C1—C2	120.9 (14)	C7—C10—H10B	110.8
O1—C1—C2	115.6 (13)	H10A—C10—H10B	108.9
C1—C2—N1	108.0 (11)	C2—N1—C5	106.4 (11)
C1—C2—C3	113.8 (14)	C2—N1—H1A	110.5
N1—C2—C3	105.4 (12)	C5—N1—H1A	110.5
C1—C2—H2	109.8	C2—N1—H1B	110.5
N1—C2—H2	109.8	C5—N1—H1B	110.5
C3—C2—H2	109.8	H1A—N1—H1B	108.6
C4—C3—C2	105.3 (13)	C8—N2—C7	107.8 (12)
C4—C3—H3A	110.7	C8—N2—H2A	110.2
C2—C3—H3A	110.7	C7—N2—H2A	110.2
C4—C3—H3B	110.7	C8—N2—H2B	110.2
C2—C3—H3B	110.7	C7—N2—H2B	110.2
H3A—C3—H3B	108.8	H2A—N2—H2B	108.5
C5—C4—C3	105.7 (15)	C1—O1—Hg1	138.6 (10)
C5—C4—H4A	110.6	C1—O1—Hg2	99.7 (10)
C3—C4—H4A	110.6	Hg1—O1—Hg2	121.1 (4)
C5—C4—H4B	110.6	C6—O3—Hg3	100.3 (10)
C3—C4—H4B	110.6	C6—O3—Hg2	146.2 (12)
H4A—C4—H4B	108.7	Hg3—O3—Hg2	113.5 (5)
C4—C5—N1	103.2 (14)	Cl2—Hg1—Cl1	168.25 (18)
C4—C5—H5A	111.1	Cl2—Hg1—O1	94.4 (3)
N1—C5—H5A	111.1	Cl1—Hg1—O1	94.2 (3)
C4—C5—H5B	111.1	Cl3—Hg2—Cl4	166.19 (18)
N1—C5—H5B	111.1	Cl3—Hg2—O1	93.9 (3)
H5A—C5—H5B	109.1	Cl4—Hg2—O1	94.9 (3)
O3—C6—O4	127.5 (16)	Cl3—Hg2—O3	90.6 (4)
O3—C6—C7	114.5 (14)	Cl4—Hg2—O3	94.2 (4)
O4—C6—C7	117.8 (14)	O1—Hg2—O3	119.6 (4)
N2—C7—C10	104.9 (12)	Cl6—Hg3—Cl5	163.51 (18)
N2—C7—C6	109.9 (11)	Cl6—Hg3—O3	103.4 (4)
C10—C7—C6	113.7 (14)	Cl5—Hg3—O3	93.0 (4)
N2—C7—H7	109.4	Cl8—Hg4—Cl7	178.5 (3)
C10—C7—H7	109.4	Cl3—Hg2—O2	95.4 (3)
C6—C7—H7	109.4	Cl4—Hg2—O2	98.4 (3)
N2—C8—C9	103.9 (17)	O1—Hg2—O2	47.2 (3)
N2—C8—H8A	111.0	O3—Hg2—O2	72.4 (3)
C9—C8—H8A	111.0	C1—O2—Hg2	86.4 (10)
N2—C8—H8B	111.0	C6—O4—Hg3	82.7 (10)
C9—C8—H8B	111.0	C6—O4—Hg4	158.6 (11)
H8A—C8—H8B	109.0	Hg3—O4—Hg4	106.4 (3)
C8—C9—C10	103.5 (19)	Cl6—Hg3—O4	95.2 (3)
C8—C9—H9A	111.1	Cl5—Hg3—O4	95.6 (3)
C10—C9—H9A	111.1	O3—Hg3—O4	47.7 (3)
C8—C9—H9B	111.1	Cl8—Hg4—O4	95.3 (3)
C10—C9—H9B	111.1	Cl7—Hg4—O4	86.2 (3)
H9A—C9—H9B	109.0	Cl2—Hg1—Cl3^i^	98.1 (2)
C9—C10—C7	104.8 (16)	Cl1—Hg1—Cl3^i^	89.20 (19)
C9—C10—H10A	110.8	O1—Hg1—Cl3^i^	94.7 (3)
C7—C10—H10A	110.8		
			
O2—C1—C2—N1	−10 (2)	Hg1—O1—Hg2—O3	−178.1 (4)
O1—C1—C2—N1	173.3 (15)	C6—O3—Hg2—Cl3	−85 (3)
O2—C1—C2—C3	107.0 (18)	Hg3—O3—Hg2—Cl3	91.5 (6)
O1—C1—C2—C3	−70 (2)	C6—O3—Hg2—Cl4	82 (3)
C1—C2—C3—C4	−133.0 (16)	Hg3—O3—Hg2—Cl4	−101.5 (6)
N1—C2—C3—C4	−14.8 (19)	C6—O3—Hg2—O1	−179 (2)
C2—C3—C4—C5	34 (2)	Hg3—O3—Hg2—O1	−3.4 (10)
C3—C4—C5—N1	−38 (2)	C6—O3—Hg3—Cl6	91.9 (12)
O3—C6—C7—N2	−177.7 (15)	Hg2—O3—Hg3—Cl6	−85.9 (6)
O4—C6—C7—N2	−1.7 (19)	C6—O3—Hg3—Cl5	−87.8 (13)
O3—C6—C7—C10	−61 (2)	Hg2—O3—Hg3—Cl5	94.5 (6)
O4—C6—C7—C10	115.4 (17)	O1—C1—O2—Hg2	18.1 (18)
N2—C8—C9—C10	−39 (2)	C2—C1—O2—Hg2	−158.7 (15)
C8—C9—C10—C7	29 (2)	O3—C6—O4—Hg3	13.3 (19)
N2—C7—C10—C9	−8(2)	C7—C6—O4—Hg3	−162.1 (13)
C6—C7—C10—C9	−128.2 (17)	O3—C6—O4—Hg4	130 (3)
C1—C2—N1—C5	114.4 (14)	C7—C6—O4—Hg4	−45 (4)
C3—C2—N1—C5	−7.6 (16)	C1—O1—Hg2—O2	9.8 (10)
C4—C5—N1—C2	28.4 (18)	Hg1—O1—Hg2—O2	−177.2 (8)
C9—C8—N2—C7	35 (2)	C6—O3—Hg2—O2	180 (3)
C10—C7—N2—C8	−16.4 (17)	Hg3—O3—Hg2—O2	−4.0 (6)
C6—C7—N2—C8	106.1 (15)	C1—O2—Hg2—Cl3	80.4 (10)
O2—C1—O1—Hg1	168.6 (12)	C1—O2—Hg2—Cl4	−99.0 (10)
C2—C1—O1—Hg1	−15 (3)	C1—O2—Hg2—O1	−10.0 (10)
O2—C1—O1—Hg2	−21 (2)	C1—O2—Hg2—O3	169.2 (11)
C2—C1—O1—Hg2	156.4 (12)	C6—O3—Hg3—O4	7.1 (11)
O4—C6—O3—Hg3	−15 (2)	Hg2—O3—Hg3—O4	−170.7 (9)
C7—C6—O3—Hg3	160.2 (10)	C6—O4—Hg3—Cl6	−110.2 (9)
O4—C6—O3—Hg2	161.1 (15)	Hg4—O4—Hg3—Cl6	89.7 (4)
C7—C6—O3—Hg2	−23 (3)	C6—O4—Hg3—Cl5	82.2 (9)
C1—O1—Hg1—Cl2	81.9 (18)	Hg4—O4—Hg3—Cl5	−77.9 (3)
Hg2—O1—Hg1—Cl2	−87.5 (6)	C6—O4—Hg3—O3	−6.7 (10)
C1—O1—Hg1—Cl1	−106.1 (18)	Hg4—O4—Hg3—O3	−166.9 (7)
Hg2—O1—Hg1—Cl1	84.4 (6)	C6—O4—Hg4—Cl8	−25 (3)
C1—O1—Hg2—Cl3	−83.9 (11)	Hg3—O4—Hg4—Cl8	87.9 (4)
Hg1—O1—Hg2—Cl3	89.1 (6)	C6—O4—Hg4—Cl7	155 (3)
C1—O1—Hg2—Cl4	106.7 (11)	Hg3—O4—Hg4—Cl7	−92.1 (3)
Hg1—O1—Hg2—Cl4	−80.4 (5)	C1—O1—Hg1—Cl3^i^	−16.5 (18)
C1—O1—Hg2—O3	9.0 (14)	Hg2—O1—Hg1—Cl3^i^	174.0 (5)

Symmetry codes: (i) *x*+1, *y*, *z*.

### Hydrogen-bond geometry (Å, °)

**Table:**

*D*—H···*A*	*D*—H	H···*A*	*D*···*A*	*D*—H···*A*
N1—H1A···Cl7^i^	0.90	2.63	3.282 (14)	130
N1—H1B···Cl6^i^	0.90	2.40	3.290 (16)	167
N2—H2A···Cl4^ii^	0.90	2.40	3.267 (15)	163
N2—H2B···Cl5^ii^	0.90	2.60	3.234 (15)	128

Symmetry codes: (i) *x*+1, *y*, *z*; (ii) *x*−1, *y*, *z*.
